# Medical Schools

**Published:** 1852-09

**Authors:** 


*Medical Schools.*

In addition to our usual exchanges, we have received during the last month a large number of announcements from medical colleges. Some of the Schools, recognizing the principle that the demand for doctors will regulate the supply, and that it is not in their power, by placing a high tariff on medical instruction, to lessen the number of practitioners—have already reduced their lecture fees, so as to meet the pecuniary ability of students. Other colleges have been compelled to adopt a similar course, in order to maintain an existence. From the *Buffalo Medical Journal*, we learn that the Faculty of the Medical Department of the University of Buffalo have reduced their fees, offering as an apology, the course pursued by other institutions with which they are brought into competition. It seems to us that if the action of our Buffalo friends is right, no apology is necessary; if reduction of fees is wrong, it cannot be justified on the ground of competition. For ourselves, we have no interest in the matter, being identified with no medical school; but we like to see consistency. J.

